# Clinical case-control study of the proprioceptive innervation of the pharynx in patients with obstructive sleep apnea

**DOI:** 10.1016/j.bjorl.2026.101836

**Published:** 2026-06-16

**Authors:** Guilherme Leal Dantas, Maria Luzete Costa Cavalcante, Erika Ferreira Gomes, Fernanda Leal Dantas Sales Pimentel, Matheus Duarte Pimentel, Conceição da Silva Martins

**Affiliations:** a Universidade Federal do Ceará, Hospital Universitário Walter Cantídio, Neurophysiology Department, Fortaleza, CE, Brazil; b Universidade Federal do Ceará, Surgery Department, Fortaleza, CE, Brazil; c Hospital Geral de Fortaleza, Otorhinolaryngology Department, Fortaleza, CE, Brazil; d Universidade Federal do Ceará, Morphology Department, Fortaleza, CE, Brazil

**Keywords:** Obstructive sleep apnea, Pharynx, Pharyngeal muscles, Nerve endings, Immunofluorescence

## Abstract

**Objective:**

To investigate the morphological aspects of the sensory innervation of the palatoglossus, palatopharyngeus, and superior pharyngeal constrictor muscles in a control group with normal sensory function and no obstructive sleep apnea, and a group of apneic patients.

**Methods:**

Subjects consisted of 7 case patients with Obstructive Sleep Apnea (OSA) diagnosed by polysomnography and prior indication of pharyngeal surgery to treat obstructive sleep-disorder-related breathing, and 6 control patients with prior surgical indication of palatine tonsillectomy for the treatment of chronic tonsillitis or recurrent tonsillitis and without moderate/severe OSA. The specimens were collected in vivo through tonsillectomy in the control group and lateral pharyngoplasty in the case group. Histomorphometry of nerve endings was assessed using hematoxylin-eosin staining in optical microscopy, and by immunofluorescence detection of nervous structures, with detailed analysis performed using confocal microscopy.

**Results:**

In the control group, muscle spindles were visualized in the palatoglossus muscle, and free, and complex nerve endings were identified in all three muscles, including Meissner, Golgi, and Ruffini-like formations, as well as spiral-wharves nerve structures in the palatoglossus and superior pharyngeal constrictor muscles. In the case group, morphological abnormalities were observed in the sensory corpuscles, with a high prevalence of unclassifiable complex nerve formations, and a reduction in the perivascular plexus. Spiral-wharves nerve formations were observed in all muscles of the apneic patients. The superior pharyngeal constrictor muscle was the most affected in the case group, showing a lower variety of nerve formations.

**Conclusion:**

In OSA, the complex arrangement of the human pharynx is impaired, with inflammation and distortion of the nervous formations, potentially impairing the pharyngeal function and contributing to the symptoms related to this disease.

**Level of evidence:**

Level 3.

## Highlights

• Pharyngeal muscles present a complex and diverse histological structure.
• In OSA there is damage to the pharyngeal muscles, mainly due to inflammation.
• In OSA there is reduced diversity of complex nerve endings in pharyngeal muscles.
• Superior Pharynx Constrictor muscle is potentially the most impaired in OSA.

## Introduction

The human pharynx comprises a series of muscles whose functional integration is essential for many complex functions, such as swallowing, speech, and breathing. From a respiratory standpoint, the primary purpose of these muscles is to keep the airway open.^1^, ^2^, ^3^ This activity is often decreased during sleep, narrowing the airway and increasing its propensity for collapse.^3^, ^4^, ^5^ Among the various conditions that affect this structure, Obstructive Sleep Apnea (OSA) is one of the most prevalent.^4^

OSA is a common disease, characterized by recurrent collapses of the pharyngeal region during sleep, which results in a substantial reduction in airflow (apnea or hypopnea), progressing to hypoxemia and hypercapnia.^6^^,^^7^ Due to the mechanisms of intermittent hypoxia, intrathoracic pressure oscillation, and sleep fragmentation, OSA can cause chronic diseases in multiple organs.^8^ In OSA, there is a correlation between local inflammation and pharyngeal denervation, combined with sensory damage, factors that reduce the effectiveness of pharyngeal protective reflexes.^6^^,^^7^^,^^9^

The motor innervation of the human pharyngeal muscles, including regional variations in their density, is well known.^10^ However, studies on sensory innervation are scarce and focus on intraepithelial sensory fibers of the pharyngeal mucosa.^11^^,^^12^ Furthermore, the relationship between OSA and morphological changes in these nerve groups still requires further clarification, with authors exploring the possibility that changes in the proprioceptive innervation of the pharynx, especially mechanosensory innervation, may contribute to OSA.

The objective of this study is to describe the histological and immunohistochemical characteristics of pharyngeal proprioceptive innervation in patients with OSA

## Methods

This study was approved by the local ethics committee (protocol numbers: 1,645,613 and 3,753,125) and followed the principles of the Declaration of Helsinki guidelines on human studies.

This is a prospective case-control study, involving the analysis of the proprioceptive innervation of the Palatoglossus Muscle (PGM), Palatopharyngeus Muscle (PPM), and Superior Pharyngeal Constrictor Muscle (SPCM), obtaining two groups of patients: a Control group with six non-apneic adult patients with normal sensory function, undergoing palatal tonsillectomy, and a Case group with seven apneic adult patients with prior surgical indication of pharyngoplasty to treat OSA.

### Control group

For the control group, individuals of both sexes were included in a screening carried out at an Otorhinolaryngology outpatient clinic among patients with previous surgical indications for palatal tonsillectomy for chronic tonsillitis or recurrent tonsillitis.

Exclusion criteria were the presence of neuromuscular disease; physical examination suggestive of changes in pharyngeal sensitivity; the presence of high risk of OSA when assessed using the STOP-BANG and Berlin scores^13^, ^14^, ^15^, ^16^; changes suggestive of OSA (apnea/hypopnea index greater than 5) in Peripheral Arterial Tonometry (PAT) using the Watch-PAT device.^17^

At the end of this screening, in the Control group, five female individuals and one male individual with ages ranging between 22- and 35-years old were analyzed.

### Case group

In the case group, only male individuals, aged between 18- and 41-years old were studied. All presented OSA, diagnosed by polysomnography, BMI < 35 kg/m^2^, and previous indication for pharyngeal surgery to treat obstructive sleep-disorder-related breathing. Individuals with neuromuscular diseases, maxillomandibular deformities, and untreated hypothyroidism or with treatment started less than a year before the therapeutic proposal, individuals using medications that act on the central nervous system and patients who had previous oropharyngeal surgeries were excluded.

### Questionnaires

Participants in the control group were assessed for OSA risk using the STOP-BANG (Snoring, Tiredness, Observed apnea, high blood pressure, Body mass index, Age, Neck circumference, and Gender) questionnaire, and by the Berlin questionnaire.^13^, ^14^, ^15^, ^16^ These were applied by a single researcher (G.L.D.) to reduce interindividual variability in judgment.

### OSA assessment

In patients of the control group who presented a low risk of OSA after applying the described questionnaires, a sleep examination was performed using the Watch-PAT equipment, which includes Peripheral Arterial Tonometry (TAP), pulse oximetry, actigraphy, snoring sensors and position, and pulse frequency. These data allow the staging of sleep into wakefulness, light sleep, deep sleep, and REM sleep, with an assessment of the severity of OSA.^17^^,^^18^

### Assessment of pharyngeal sensitivity

Presumption of normal pharyngeal sensitivity occurred during the oropharyngoscopy of the otorhinolaryngological physical examination, considered positive in the presence of contraction and/or nausea reflex when touching the anterior and posterior tonsillar pillars and the posterior wall of the oropharynx with a tongue depressor.

### Sample collection

Samples of the PGM, PPM, and SPCM from the Control group were obtained during the conventional palatal tonsillectomy procedure. Extracapsular dissection of the palatine tonsils was performed bilaterally with cold forceps and cauterization with bipolar cautery for hemostasis of the tonsillar pockets.

All patients in the Case group underwent the modified expander pharyngoplasty surgical procedure for the treatment of OSA.^19^

Surgical procedures in both groups were performed under general anesthesia, with tracheal intubation, in horizontal dorsal decubitus with cervical hyperextension (Rose position). The specimens were obtained intraoperatively and consisted of fragments approximately 1 cm long and 0.3 cm wide and thick. Sample removal was an additional procedure to the previously indicated surgery, with standardization of the location for collecting samples from the three muscles in all individuals, opting for the patient's right side. All procedures were performed by the same surgeon, and there were no surgical complications.

### Sample preparation

The samples were placed in containers with a 4% paraformaldehyde solution, where they remained refrigerated for 48–72 hours. Then, the pieces were transferred to containers with 20% sucrose solution and stored in a freezer at −70 °C until they were cut and stained.

### Hematoxylin and eosin staining

The sample sections were placed on Immunoslide® slides (Easypath®, Brazil) and stained with Harris hematoxylin and yellowish eosin immediately after cutting to evaluate the preservation and integrity of the tissues.

### Immunohistochemistry (IH)

Slide preparation followed the immunofluorescence method with primary antibody PGP 9.5 and secondary antibody Alexa fluor 488, described by Jew and collaborators.^20^ The sections were washed four times for 15 min with a cold 0.1 moL phosphate-buffered saline solution (0.1 M PBS, Laborclin®, Brazil) containing 3% Triton X-100 (TX-100, Inlab®, Brazil), followed by incubation for two hours at room temperature, with blocking solution containing 4% normal goat serum (Jackson Immuno Research Inc., West Grove, PA, USA), 0.25% bovine serum albumin (Inlab®, Brazil), 2% TX-100 and 0.1 M PBS.

Tissues were washed for 15 min four times with ice-cold 0.1 M PBS and incubated with primary antibody for 18–20 hours at 4 °C. The primary antibody was PGP 9.5 (Thermo Fisher Scientific Inc., Rockford, IL, USA), diluted 1:200 in a solution consisting of 0.5% TX-100 in 0.1 M PBS. It was then washed four times for 15 min in 0.1 M PBS and then incubated in the dark for two hours at room temperature with the second fluorescent marker antibody Alexa Fluor 488–IgG (Thermo Fisher Scientific Inc., Rockford, IL, USA), diluted 1:200 with the primary antibody diluents. The slides were protected from light, and a series of washes were performed: twice for 15 min with cold 0.1 M TFS, once for 20 min with cold 0.05 M TFS, and once with distilled water. The slides were covered with coverslips, placed in cases, and stored in a refrigerator at −70 °C.

The specimens were then evaluated by confocal laser scanning microscopy for a detailed assessment of the nervous structures present in the studied tissues.

### Statistical analysis

Quantitative data were expressed as mean ± Standard Deviation (SD). Data distributions were verified using the Shapiro-Wilk test. Parameter data with p < 0.05 for the Shapiro–Wilk test were considered to have a non-parametric distribution. The *t*-test followed by the Wilcoxon test was also performed for non-parametric data. The non-parametric Kruskal-Wallis’ test was used followed by the Dunn’s test for multiple comparisons in the Control group.

The analyses were carried out using the SPSS software for Windows (version 20.0), and the graphs were developed using the GraphPad Prism software resources (version 8.0). For all analyses, it was considered statistically significant when p < 0.05.

## Results

### Palatoglossus muscle

In the control group, a stratified squamous epithelium was observed, with connective tissue with intense vascularization and muscle bundles, with nerve filaments in subepithelial connective tissue related to blood vessels and between muscle fibers. In the IH analysis, the control group found the presence of fine free nerve endings in different directions within the subepithelial connective tissue, in the muscular layer, parallel to the muscle fibers, and in close relation to the blood vessels.

Within the muscular layer, complex nerve endings of different sizes and shapes were also identified, such as network-like formations, non-encapsulated Ruffini-like arborized formations, a tangle of nerve fibers with a Golgi-like conical shape, Meissner-like cylindrical structures in the tissue connective tissue close to the muscles and elongated nervous structures with a spiral shape (spiral-wharves), which ran parallel to the muscle fibers. Additionally, muscle spindles were found sparsely throughout the tissue smaples.

**Fig. 1:**
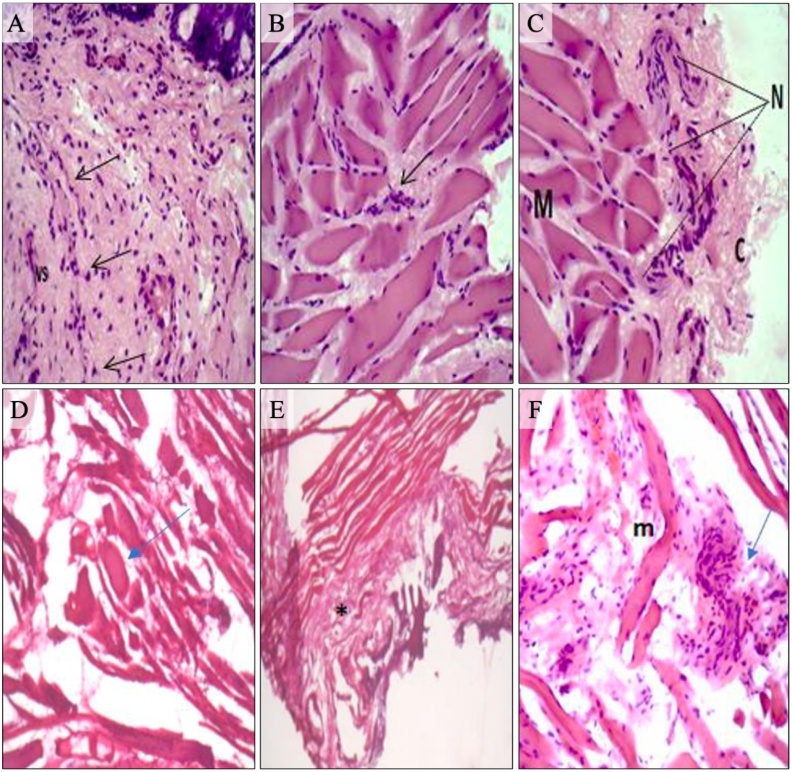
Light microscopy images illustrating histological structure in palatoglossus muscle tissues of control and case patients. (A) Control group (×200): Nerve endings (arrow) within subepithelial connective tissue. (B) Control group (×200): Nerve bundle (arrow) inside muscle fibers; (C) Control group (×200): Nervous fiber (n) within connective tissue (c) entering the muscle fibers (m). (D) Case group (×100) Compensatory muscular hypertrophy (arrow); (E) Case group (×50): Inflammatory infiltrate (star) in subepithelial space; (F) Case group (×200): Degraded nerve endings (arrow) next to muscular fibers (m).

**Fig. 2:**
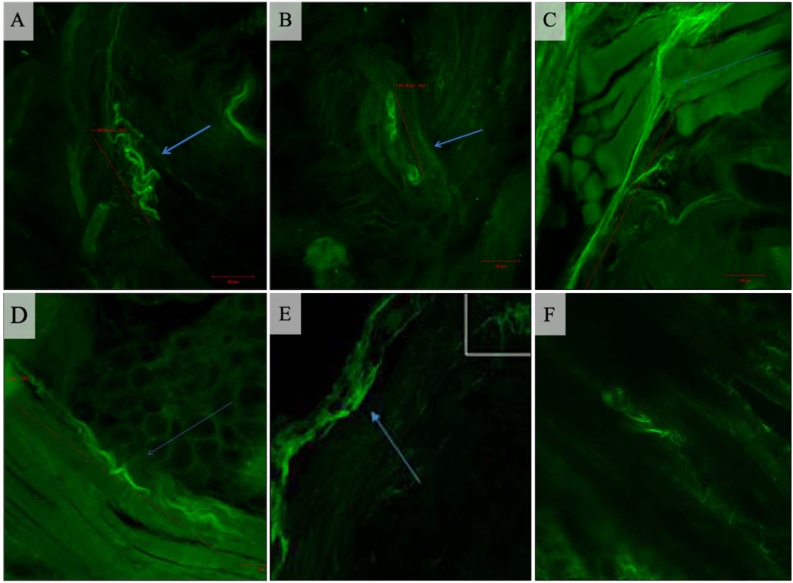
Immunofluorescence analysis of the palatoglossus muscle in the studied groups. (A) Control group (×20): *Ruffini-like* non-encapsulated formation (arrow) within the muscular layer. (B) Control group (×20): *Meissner-like* corpuscle (arrow) in connective tissue; (C) Control group (×20): Diffuse nerve bundles distributed within the muscle fibers, constituting a muscle spindle; (D) Control group (×20): Elongated nerve structures in spiral format (*spiral-wharves*) running parallel do muscle fibers. (E) Case group (×10): Subepithelial nerve fibers with progressive reduction in width running parallel to muscle fibers. (F) Case group (×20): *Spiral-wharve* nerve formation immerse in sparse muscular fibers.

In the group of patients with OSA, in turn, non-keratinized stratified squamous epithelium (parakeratosis) was observed with vacuolated seromucous glands resulting from lipid degeneration, forming epidermoid cysts within the epithelium and, eventually, epithelial invagination. An inflammatory infiltrate was observed in the subepithelial space. Furthermore, there was a clear reduction in the visualization of nerve fibers which, in addition to their lower frequency, were degenerated, with a dysmorphic pattern, in contrast to the muscles, tending to form nerve threads. Rarely, neurovascular bundles were visualized. These findings are illustrated in Fig. 1, Fig. 2.

### Palatopharyngeus muscle

Similarly to the previously described muscle, sections from patients of the control group presented a stratified squamous epithelium, connective tissue with intense vascularization in the deeper layers, many mucus-secreting acinar glands, and a muscular layer with disconnected muscle fibers. Thin and tortuous free nerve endings were noted in the epithelium, heading towards the blood vessels and, in the muscular layer, parallel to the muscle fibers, with Golgi-like and Ruffini-like complex nerve endings in the layer muscle.

**Fig. 3:**
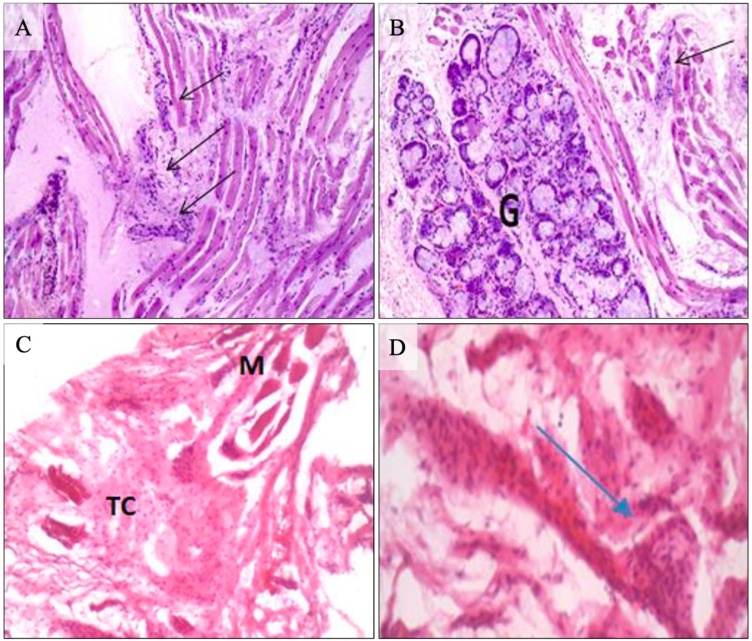
Light microscopy images illustrating histological structure in palatopharyngeus muscle tissues of control and case patients. (A) Control group (×100): Nerve endings (arrows) between muscular fibers. (B) Control group (×100): Mucossecreting acinar Glands (G) and nerve bundle (arrow) within the muscle fibers. (C) Case group (×100): Sparse and disconnected Muscle fibers (M) within Connective Tissue (TC). (D) Case group (×200): Complex neural structure (arrow) within connective tissue.

**Fig. 4:**
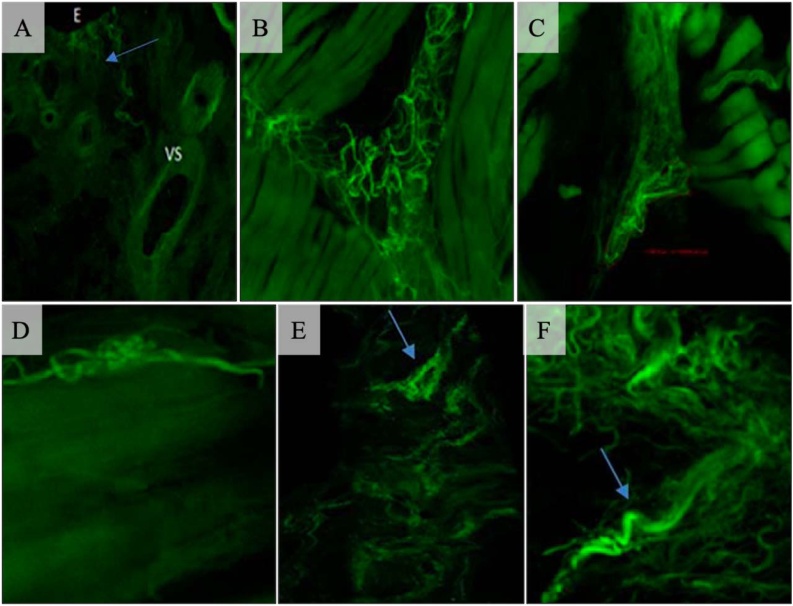
Immunofluorescence analysis of the palatopharyngeus muscle in the studied groups. (A) Control group (×10): Thin and tortuous free nerve endings (arrow) in the epithelial layer running towards blood Vessels (VS). B. Control group (×20): Complex *Golgi-like* nerve formation; (C) Control group (×20): *Ruffini-like* non-encapsulated formation. (D) Case group (×20): Non-classifiable nerve formation. (E) Case group (×10): *Golgi-like* nerve formation. (F) Case group (×20): *Spiral-wharves* nerve formation.

In the case group, it was not possible to identify the epithelium in the samples studied (Fig. 3). Sparse and disconnected muscle fibers were visualized. In the connective tissue, an inflammatory process was observed. The rare nerve structures were displayed as sensory corpuscles or in neurovascular bundles through complex neural structures close to blood vessels within the connective tissue (Fig. 4).

### Superior pharyngeal constrictor muscle

**Fig. 5:**
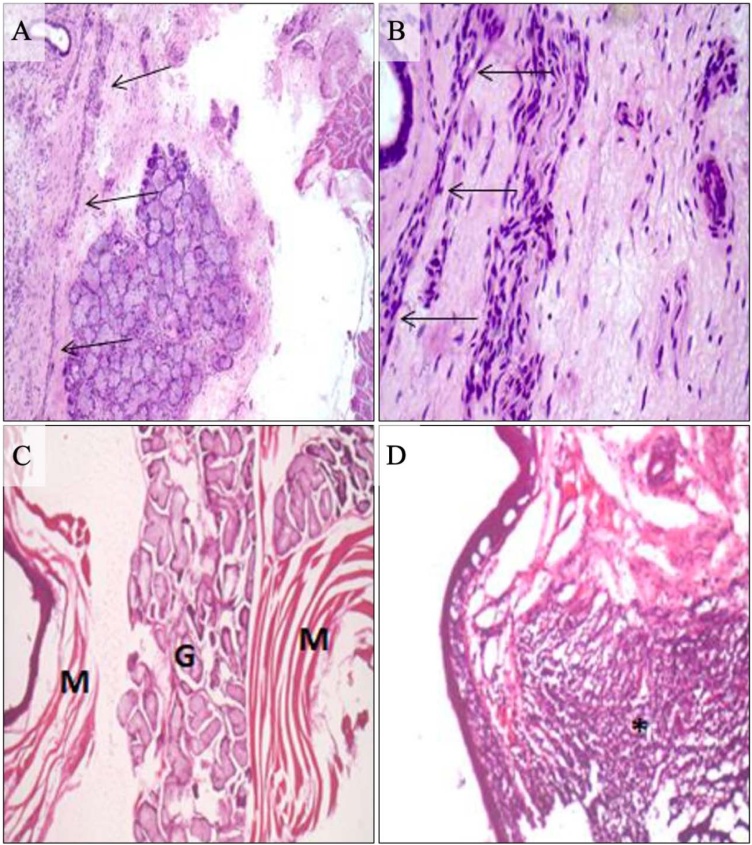
Light microscopy images illustrating histological structure in superior pharyngeal constrictor muscle tissues of control and case patients. (A) Control group (×50): Long nerve strip (arrows) in the subepithelial connective tissue. (B) Control group (×200): Higher magnification of the structure of the nerve fiber within the connective tissue. (C) Case group (×50): Mucoacinar Glands (G) interposed between the Muscle fibers (M) in the connective tissue. (D) Case group (×100): Intense inflammatory process in the submucosa.

In samples from the control group, we visualized non-keratinized stratified squamous epithelium, connective tissue with vessels and nerve endings, long nerve threads in the subepithelial connective tissue, and a cluster of mucosecretory acinar glands. Free and complex nerve endings were also observed, maintaining an intimate relationship with seromucosal glands. In the deepest layers of the lamina propria, large nerve bundles were identified, measuring more than 200 μm in thickness (Fig. 5).

In the case group, non-keratinized stratified squamous epithelium was also observed. As in the other muscles studied, asymmetrical muscle fibers were found, with different calibers and diameters, indicating muscle atrophy. Mucoacinar glands were observed interposed between the muscle fibers in the connective tissue.

**Fig. 6:**
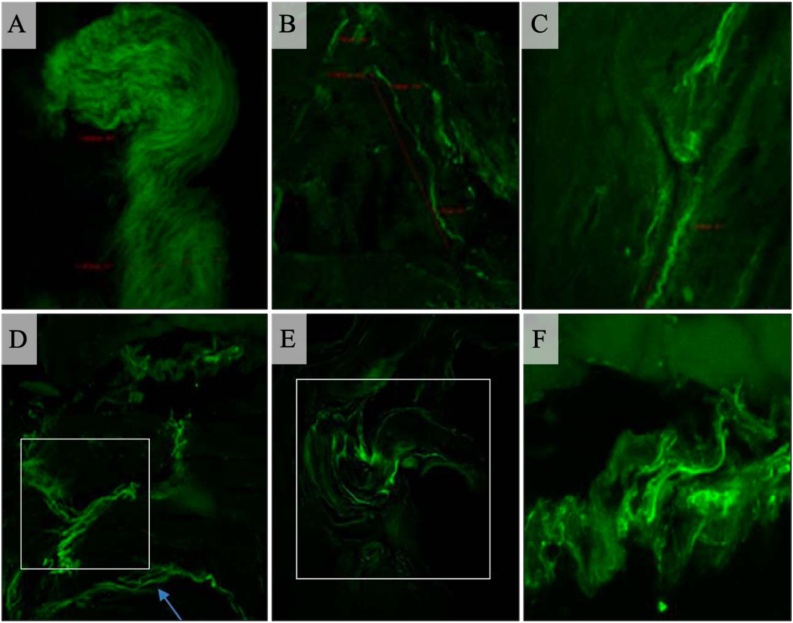
Immunofluorescence analysis of the superior pharyngeal constrictor muscle in the studied groups. (A) Control group (×20): Nerve bundle. (B) Control group (×20): Free nerve endings in proximity to seromucous glands. (C) Control group (×20): *Spiral-wharves* nerve formations. (D) Case group (×20): *Network-type* nerve formation (within the rectangle) and free nerve ending beneath (arrow). (E) Case group (×10): Complex non-classifiable nerve ending. (F) Case group (×20): *Ruffini-like* sensorial corpuscle.

It is noteworthy that among the three muscles of the case group analyzed, the SPCM demonstrated the smallest variety of nerve formations, in contrast to what was observed in the Control group. The same pattern of dispersion of muscle fibers of the PGM was evident. The sensory corpuscles did not demonstrate the same clarity and richness of details observed in the Control group (Fig. 6).

Detailed data on the nerve endings found in each muscle group studied are described in Table 1.

**Table 1:** Distribution of nerve endings between the palatoglossus, palatopharyngeus, and superior pharyngeal constrictor muscles.

Nerve ending	Palatoglossus		Palatopharyngeus		Superior pharyngeal constrictor muscle	
	Control group	Case group	Control group	Case group	Control group	Case group
Free Nerve Endings	+	+	+	+	+	+
Ruffini-like formation	+	+	+	–	+	+
Golgi-like formation	+	–	+	+	–	–
Meissner-like formation	+	–	–	+	–	–
Oval complex formation	+	–	–	–	+	–
Spiral-wharves	+	+	–	+	+	+
Network formation	+	+	+	+	–	+
Unclassifiable complex formation	–	–	–	+	+	+
Nerve Bundles	+	+	+	+	+	–
Nerve Fascicles	–	–	–	–	+	–
Muscle Spindles	+	–	–	–	–	–

## Discussion

The coordinated neuronal regulation of the pharyngeal muscles is fundamental for the adequate control of the size and resistance of the upper airway. The pathophysiological mechanism for upper airway collapse in patients with OSA has not yet been completely elucidated, though several studies have shown the occurrence of pharyngeal neuropathy in patients with OSA.^1^^,^^2^^,^^6^^,^^9^^,^^11^

Considering the participation of the lateral pharyngeal wall in the collapse of the upper airway during an apnea episode, there is considerable relevance in studying the proprioceptive sensory innervation of the muscles that compose it, notably the PGM, PPM, and SPCM.^21^, ^22^, ^23^

In a recent study, Askar analyzed the relevance of the PPM, highlighting its participation in the pharyngeal shortening, and in the movement of both the soft palate and the hyolaryngeal complex.^24^ This muscle would be activated in response to negative pressure, presenting an important role in the patency of the upper airway, making it a subject for studies focused on the physiopathology of the OSA, which have showed that modifications in the muscular fibers or in the sensory innervation of the PPM may contribute to an inadequate upper airway collapse in this condition.^24^^,^^25^

Histological evaluation in the Control group demonstrated that the various layers had not undergone important changes, guaranteeing the integrity of the tissues analyzed. The presence of inflammatory processes was also not found in the samples studied, thus ensuring that chronic inflammation of the tonsils did not interfere with the analysis of the sensitive nerve endings of the tissues analyzed. On the other hand, in the case group, the presence of intense vacuolation of the glands resulting from lipid degeneration, indicating the occurrence of stress and aggressive factors typical of obstructive sleep events, as well as changes in the architecture of the muscle tissue, such as compensatory muscle atrophy and hypertrophy, were characteristics related to the pathophysiology of OSA, as illustrated by other authors.^6^^,^^7^^,^^21^

The presence of inflammation was also found in the three muscles studied, highlighting the presence of Langerhans cells in the palatopharyngeal muscle and an intense inflammatory process in the SPCM, where the greatest variability of inflammatory cells was observed, such as lymphohistiocytic infiltrate. It is known that chronic inflammation can play an important role in the pathogenesis of OSA, corroborating the studies by Boyd et al.^26^

Specialized sensory corpuscles were noted in the case group, but were smaller, some with an atrophic or hypotrophic appearance, suggesting an anatomical distortion caused by OSA. The greatest variety of these structures was observed in the palatopharyngeus muscle, and, among the classic corpuscular formations, Ruffini-type nerve endings were the most frequently found. While the SPCM demonstrated a very robust and diverse innervation pattern in the control group, the same was not observed in the case group, with this one appearing to be the most affected muscle among the three studied. In view of the crucial importance of this muscle for the physiology of the pharynx and the maintenance of lateral wall stability during sleep, this qualitative sensory impairment appears to have significant relevance in understanding the pathophysiology of OSA.

The most important and promising structures identified in the research were certainly the spiral-wharves nerve formations, identified in the Control group as long nerve structures that ran parallel to the muscle fibers of the PGM and SCPM. These structures were also found in all muscles studied in the case group. Due to their arrangement, it is possible to hypothesize that these nerve formations are capable of shortening or lengthening, depending on the activities of the pharyngeal muscles, to trigger reflexes that adapt the tone of the pharyngeal muscles to a specific situation. These structures were rarely described in the literature, highlighting studies involving the lateral pharyngeal wall and vocal folds of newborns.^27^^,^^28^ In a comparative study of sensory innervation of the pharynx between patients with and without OSA, spiral-wharve nerve formations were also found in both groups, as in the present study.^25^ It is believed that these formations, due to their strategic conformation, are the main candidates for replacing muscle spindles in the proprioceptive function of the pharynx. Additionally, muscle spindles, structures deemed responsible for the proprioceptive function in skeletal muscles, were found only in the PGM what could be explained by its controversial muscular origin, since it is considered by several authors an extrinsic muscle of the tongue.

The great variety and complexity of the nerve formations found in the Control group of the present research can be explained by the methodology applied and by the fact that the study also included the PGM and palatopharyngeal muscles, while the main studies that addressed proprioceptive sensory innervation of the pharynx focused specifically on the SPCM.^28^^,^^29^ In the case group, on the other hand, this scope and variety of nerve formations were not observed. In this group, a pattern of distribution of nerve fibers was observed, notably concentrated in the periphery and most of the vascular plexus was absent or severely reduced, in agreement with the article of Carlos and collaborators.^25^ Another interesting finding was the high prevalence of unclassifiable complex nerve formations in relation to the Control group. It is believed that the anatomical distortion of structures caused by OSA created difficulty in identifying the more classic sensory corpuscles, which, when assuming a dysmorphic pattern, were included in this type of classification of mechanoreceptors.

### Limitations

The study presents, however, some limitations that need to be described. The research was carried out in vivo, thus limiting more extensive tissue dissections. Furthermore, the number of samples evaluated was limited, and only one neuronal antibody was used. Another limitation of the study is related to the gender bias in the analysis of the results. The Control group was made up mainly of women, while the case group was exclusively made up of men, something that may affect the comparative analyses between the two groups.

## Conclusion

In summary, the human pharynx muscles present, physiologically, an intricate morphological structure, with diverse complex nerve endings formations. In OSA, this arrangement is impaired, with inflammation and distortion of the nervous formations, potentially impairing the pharyngeal function and contributing to the symptoms related to this disease.

## ORCID ID

Guilherme Leal Dantas: 0000-0001-9656-5003

Maria Luzete Costa Cavalcante: 0000-0002-3363-6916

Erika Ferreira Gomes: 0000-0002-8165-4609

Fernanda Leal Dantas Sales Pimentel: 0000-0002-2416-3993

Matheus Duarte Pimentel: 0000-0001-9833-3943

Conceição da Silva Martins: 0000-0001-8710-1856

## Author’s contributions

Conceptualization: Dantas GL, Cavalcante MLC, Gomes EF, Pimentel FLDS, Pimentel MD, Martins CS.

Data curation: Dantas GL, Cavalcante MLC, Gomes EF, Pimentel FLDS, Pimentel MD, Martins CS.

Investigation: Dantas GL, Cavalcante MLC, Gomes EF, Martins CS.

Methodology: Dantas GL, Cavalcante MLC, Martins CS, Pimentel MD.

Resources: Dantas GL, Cavalcante MLC, Gomes EF, Martins CS.

Software: Dantas GL, Cavalcante MLC, Pimentel MD.

Supervision: Cavalcante MLC, Gomes EF.

Writing-original draft: Dantas GL, Cavalcante MLC, Gomes EF, Pimentel FLDS, Pimentel MD, Martins CS.

Writing-review & editing: Dantas GL, Cavalcante MLC, Gomes EF, Pimentel FLDS, Pimentel MD, Martins CS.

## Consent for publication

The manuscript is not submitted for publication or consideration elsewhere.

## Ethics approval and consent to participate

This study was conducted in accordance with the declaration of Helsinki. This study was conducted with approval from the Ethics Committee of The Federal University of Ceará. (Protocol nº 1,645,613 and 3,753,125). Written informed consent was obtained from all participants.

## Funding

No funding was provided for this study.

## Data availability statement

All data generated or analyzed during this study are included in this published article.

## Declaration of competing interest

The authors declare no conflicts of interest.

## References

[1] Van Lunteren E. (1993). Muscles of the pharynx: structural and contractile properties. Ear Nose Throat J..

[2] Palombini L.O. (2010). Fisiopatologia dos distúrbios respiratórios do sono. J Bras Pneumol.

[3] Malhotra A., Huang Y., Fogel R. (2006). Aging influences on pharyngeal anatomy and physiology: the predisposition to pharyngeal collapse. Am J Med..

[4] Isono S., Remmers J.E., Tanaka A., Sho Y., Sato J., Nishino T. (1997). Anatomy of pharynx in patients with obstructive sleep apnea and in normal subjects. J Appl Physiol (1985).

[5] Remmers J.E., DeGroot W.J., Sauerland E.K., Anch A.M. (1978). Pathogenesis of upper airway occlusion during sleep. J Appl Physiol Respir Environ Exerc Physiol..

[6] Lv R., Liu X., Zhang Y. (2023). Pathophysiological mechanisms and therapeutic approaches in obstructive sleep apnea syndrome. Signal Transduct Target Ther..

[7] Eckert D.J., Malhotra A. (2008). Pathophysiology of adult obstructive sleep apnea. Proc Am Thorac Soc..

[8] Salman L.A., Shulman R., Cohen J.B. (2020). Obstructive sleep apnea, hypertension, and cardiovascular risk: epidemiology, pathophysiology, and management. Curr Cardiol Rep..

[9] White D.P. (2017). Advanced concepts in the pathophysiology of obstructive sleep apnea. Adv Otorhinolaryngol..

[10] Yoshida Y., Tanaka Y., Hirano M., Nakashima T. (2000). Sensory innervation of the pharynx and larynx. Am J Med..

[11] Cole C.L., Yu V.X., Perry S. (2023). Healthy human laryngopharyngeal sensory innervation density correlates with age. Laryngoscope..

[12] Foote A.G., Thibeault S.L. (2021). Sensory innervation of the larynx and the search for mucosal mechanoreceptors. J Speech Lang Hear Res..

[13] Pivetta B., Chen L., Nagappa M. (2021). Use and performance of the STOP-Bang questionnaire for obstructive sleep apnea screening across geographic regions: a systematic review and meta-analysis. JAMA Netw Open..

[14] Chung F., Abdullah H.R., Liao P. (2016). STOP-Bang questionnaire: a practical approach to screen for obstructive sleep apnea. Chest..

[15] Chung F., Yegneswaran B., Liao P. (2008). Validation of the Berlin Questionnaire and American Society of Anesthesiologists checklist as screening tools for obstructive sleep apnea in surgical patients. Anesthesiology..

[16] Senaratna C.V., Perret J.L., Matheson M.C. (2017). Validity of the Berlin questionnaire in detecting obstructive sleep apnea: a systematic review and meta-analysis. Sleep Med Rev..

[17] Choi J.H., Kim E.J., Kim Y.S. (2010). Validation study of portable device for the diagnosis of obstructive sleep apnea according to the new AASM scoring criteria: Watch-PAT 100. Acta Otolaryngol..

[18] Pinto J.A., Godoy L.B.M., Ribeiro R.C., Mizoguchi E.I., Hirsch L.A.M., Gomes L.M. (2015). Accuracy of peripheral arterial tonometry in the diagnosis of obstructive sleep apnea. Braz J Otorhinolaryngol..

[19] Ulualp S.O. (2014). Modified expansion sphincter pharyngoplasty for treatment of children with obstructive sleep apnea. JAMA Otolaryngol Head Neck Surg..

[20] Jew J.Y., Berger E.J., Berger R.A., Lin Y.T. (2003). Fluorescence immunohistochemistry and confocal scanning laser microscopy: a protocol for studies of joint innervation. Acta Orthop Scand..

[21] Tsai Y.-J., Ramar K., Liang Y.-J. (2013). Peripheral neuropathology of the upper airway in obstructive sleep apnea syndrome. Sleep Med Rev..

[22] Saboisky J.P., Butler J.E., Luu B.L., Gandevia S.C. (2015). Neurogenic changes in the upper airway of obstructive sleep apnoea. Curr Neurol Neurosci Rep..

[23] Guilleminault C., Ramar K. (2009). Neurologic aspects of sleep apnea: is obstructive sleep apnea a neurologic disorder?. Semin Neurol..

[24] Askar S.M. (2024). The palatopharyngeal muscle in otolaryngology practice: an anatomical-based surgical report. Eur Arch Otorhinolaryngol..

[25] De Carlos F., Cobo J., Macías E. (2015). Reduced innervation in the human pharynx in patients with obstructive sleep apnea. Histol Histopathol..

[26] Boyd J.H., Petrof B.J., Hamid Q., Fraser R., Kimoff R.J. (2004). Upper airway muscle inflammation and denervation changes in obstructive sleep apnea. Am J Respir Crit Care Med..

[27] Gonçalves da Silva Leite J., Costa Cavalcante M.L., Fechine-Jamacaru F.V. (2016). Morphology of nerve endings in vocal fold of human newborn. Int J Pediatr Otorhinolaryngol..

[28] De Carlos F., Cobo J., Macías E. (2013). The sensory innervation of the human pharynx: searching for mechanoreceptors. Anat Rec (Hoboken)..

[29] Arias García C. (2012). https://digibuo.uniovi.es/dspace/handle/10651/4070.

